# ZnO-embedded S-doped g-C_3_N_4_ heterojunction: mediator-free Z-scheme mechanism for enhanced charge separation and photocatalytic degradation

**DOI:** 10.1039/d0ra04642f

**Published:** 2020-07-29

**Authors:** Periyathambi Kalisamy, Mathiazhagan Lallimathi, Mathiazhagan Suryamathi, Baskaran Palanivel, Munusamy Venkatachalam

**Affiliations:** Department of Chemistry, Government Arts College for Men Krishnagiri TamilNadu-635 001 India venkatphd06@gmail.com; Department of Chemistry, Periyar University Salem TamilNadu-636 011 India; Department of Physics and Nanotechnology, SRM Institute of Science and Technology Kancheepuram TamilNadu-603 203 India baskaranpj1993@gmail.com

## Abstract

The design of UV-visible light active photocatalysts for organic pollutant removal is a challenging task. Herein, we have developed an LED light active ZnO-embedded S-doped g-C_3_N_4_ (SCN) heterojunction by a facile sol–gel assisted calcination method. The heterojunction between ZnO and SCN nanoparticles generates a Z-scheme photocatalyst, which helps to separate the photo-induced charge carriers in the opposite direction, and is beneficial for more visible light absorption for photocatalytic dye degradation. The composite heterojunction shows better photocatalytic redox in comparison with pristine nanomaterials. The enhanced degradation efficiency is attributed to the high production rate of ˙OH (hydroxyl) radicals during the photocatalysis process, which is analyzed by the TA test and elemental trapping experiment. Hence, we hope that this Z-scheme heterojunction provides a new way to develop UV-visible light active photocatalysts for environmental remediation applications.

## Abstract 2

We have developed the LED light active ZnO-embedded S-doped g-C_3_N_4_ (SCN) mediator free Z-scheme heterojunction photocatalyst for effective organic pollutant degradation.

## Graphical abstract

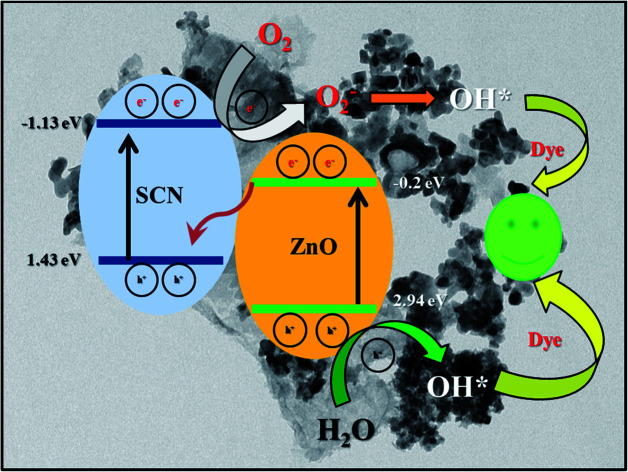

## 1. Introduction

In recent years, unpredictable climate change and human population increase have caused a crisis in pure water availability for humans and wildlife. Moreover, overconsumption of water due to increasing industrial sectors has caused the depletion of freshwater. So, it is urgent to renew wastewater and reuse processes to ensure sustainable growth and management. Several purification methods such as adsorption and coagulation, have been developed and tested on wastewater to remove contaminants. However, most of the methods are not suitable for recalcitrant pollutants. Moreover, these conventional methods produce secondary harmful pollutants because the organic dyes are changed from the liquid phase to the solid phase. Therefore, it is necessary to develop purification methods to degrade the low biodegradable and highly chemically stable pollutants. In this way, heterogeneous semiconductor-based photocatalysis plays an important role in the removal of organic, inorganic, and microbial pollutants. In heterogeneous photocatalytic reactions, the light-absorbing catalyst is put into contact with the target pollutants in either a solution phase or gas phase. Moreover, the use of heterogeneous photocatalysts does not produce secondary impurities.^1–3^ Several semiconductors have been tested for photocatalytic organic pollutant removal. Among them, ZnO is one of the most available photocatalysts, due to its abundant in nature, low cost, non-toxic, versatility in preparation methods, suitable redox potential, and physio-chemical stability. This n-type semiconductor has a wide bandgap of around 3.2 eV and large excitation binding energy of 60 meV at room temperature. In addition to this, ZnO possesses antifouling and antibacterial properties, which makes it a promising material for organic, inorganic, and microbial pollutant removal processes. However, low solar light utilization due to the large bandgap, low quantum efficiency, and fast electron–hole recombination are considered as bottlenecks for practical implementation.^4–8^ In order to improve the photocatalytic efficiency of ZnO, several strategies have been developed, such as doping with metal and non-metals, control of the morphology, and making the heterojunction using other semiconducting materials. These methods help to enhance the light absorption ability and to improve the photocatalytic activity of ZnO. Semiconductor materials coupling is the most promising route to separate the photo-induced electron–hole pairs because the energy potential of the valance and conduction bands (CBs) are different to each other. This heterojunction formation helps to suppress the electron–hole pair recombination and enhances the photocatalytic efficiency.^9^ Moreover, the coupling of low-bandgap semiconductors can sensitize the ZnO to visible light, and promote charge-carrier separation.^10^

Based on this, coupling ZnO with graphitic-carbon nitride (g-C_3_N_4_) is considered to be the most predominant route to improve visible light absorption in order to enhance the photocatalytic activity.^11,12^ Graphitic carbon nitrides are visible-light-driven photocatalysts which have been paid great attention in the field of photocatalysis due to their low cost, facile preparation, high chemical stability and thermal stability, eco-friendliness and moderate bandgap of 2.7 eV. Moreover, the graphitic phase C_3_N_4_ is the most stable form compared to all other allotrope forms at ambient conditions. The nitrogen presented in the carbon nitride helps to harvest more visible light due to its high potential energy and low electronegativity in comparison with oxygen.^13–16^ The non-metal doping is the promising route to tune the energy band structure of the semiconductor materials.^17^ The doping of heteroatoms such as P, S, B, and C, *etc.* into the g-C_3_N_4_ network further shrinks the bandgap, which helps to harvest more visible light for the photocatalytic process. Among these, sulfur addition may be preferable for effective photocatalytic activity: sulfur doping is beneficial for electron mobility and conductivity, which helps to reduce the electron–hole recombination rate during the photocatalysis process.^18–22^ Moreover, the high electronegativity of the sulfur atom helps to narrow the bandgap of the g-C_3_N_4_. Besides, sulfur doping enhances the optical properties of g-C_3_N_4_ in terms of light absorption intensity and absorption range.^23,24^

Based on the above considerations, we have prepared ZnO-embedded S-doped g-C_3_N_4_ (SCN) heterojunctions by a single step approach. To the best of our knowledge there are no reports for a single-step sol–gel followed by calcination process for direct Z-scheme ZnO/SCN heterojunction preparation. The prepared heterojunction photocatalysts were analyzed by various characterization methods and were subjected to organic pollutant degradation under commercial indoor LED light irradiation. The pollutant degradation report demonstrated that heterojunction photocatalysts exhibit the best catalytic activity in comparison with pure materials. The radicals scavenging test was used to predict the reactive radicals for the Z-scheme photocatalytic degradation process. In a direct solid-state Z-scheme process, the electrons are migrated through interfacial contact between the photo-active semiconductors without any electron mediator. In the Z-scheme photocatalysis process, the two-step photo-excitation process was involved, in which two different semiconductors perform the reduction and oxidation reaction.^25,26^ Especially, the spatial separation of oxidation and reduction centers is achievable in such a two-step photoexcitation system, which can minimize the undesirable back-reaction to enhance the overall photo-conversion efficiency.^27^ In this typical Z-scheme process, the electrons in the CB of one semiconductor transferred to another semiconductor’s valance band through an inter cross-section without any electron shielding problem.^15^ Most importantly, the reduction and oxidation reactions occur at the heterogeneous photocatalysts with more negative CB values and more positive valance band values, respectively. Moreover, this type of Z-scheme heterojunction, with strong oxidation and reduction potential is useful for boosting the overall photo-conversion efficiency.^26^ In this way, the electron and holes are separated in opposite directions and help to enhance the photocatalytic redox process.

## 2. Experimental

### 2.1. Synthesis of nanoparticles

The facile sol–gel calcination process followed was used to synthesize the photocatalysts. In the typical process, 1 mmol of zinc nitrate hexahydrate and citric acid were dissolved in 5 ml of ethanol. Then the optimised amount of thiourea was added to the solution and stirred until the gel was formed. Then the obtained gel was dried in a hot air oven at 80 °C for 3 h. The dried powder was ground in an agate mortar and calcined to 550 °C for 2 h. The prepared nanoparticles were washed with ethanol and de-ionized water five times to remove any residual adhesive carbon. The obtained powder was named ZnO/SCN. The measured weight percentage of ZnO in the composite was 61% and SCN was 39%. The above mentioned procedure was followed to synthesize the pure ZnO without the addition of thiourea.

The S-doped g-C_3_N_4_ was prepared by direct polymerization of thiourea. In the typical process, 3 g of thiourea was taken in a crucible and calcined to 550 °C for 2 h. For comparison purposes, bare g-C_3_N_4_ (BCN) was prepared using melamine.

### 2.2. Characterizations

The crystalline nature of the prepared photocatalysts was analyzed by a BRUKER USA D8 Advance, Davinci powder X-ray diffractometer. The morphological properties were analyzed by FESEM (JEOL, JSM-7100F) and HRTEM (JEOL, JSM-2100plus) microscopes. The optical properties were studied by a Shimadzu UV3600+ spectrometer (UV-DRS) and HORIBA Fluorolog (PL). The XPS analysis of the photocatalyst was carried out by PHI Versaprobe III. The FTIR analysis of the nanoparticles was studied using a SHIMADZU, IRTRACER-100 with ATR mode. The mineralization of the MB dye solution was analyzed by a TOC (Shimadzu TOC-L CPN) analyzer.

### 2.3. Photocatalytic activity

The photocatalytic activity of the prepared nanoparticles was analyzed by the degradation of methylene blue (MB) and rhodamine B (RhB) dye molecules under LED light illumination without any optical filter. In a typical photocatalysis process, 50 mg of catalyst was added to 100 ml of dye solution (10 ppm). The adsorption–desorption equilibrium dye solution was attained by stirring for 30 min with the catalyst. Afterward, the dye solution was irradiated by a 40 W LED lamp (31 619 lux) under ambient temperature and atmospheric pressure. At regular intervals of time, 3 ml of dye solution was taken out and analyzed for the remaining concentration of dye solution using a Shimadzu UV3600+ spectrophotometer. The absorption maximum at 664 nm and 554 nm was used to detect the concentration of MB and RhB, respectively. The radicals scavenging experiment was conducted with the help of BQ, EDTA, and IPA for superoxide radicals, holes, and hydroxyl radicals, respectively. To detect the production of OH radicals the terephthalic acid (TA) test was conducted:^28^ typically, 0.075 mmol of TA was dissolved in 1000 ml of water. Then 100 ml of TA solution was removed and 50 mg of catalyst was added to the solution and irradiated by LED light. At certain time intervals, 2 ml of solution was taken and analyzed by PL spectroscopy with an excitation wavelength of 315 nm. The emission peak intensity at 425 nm was recorded to detect the production of the ˙OH radical in the photocatalysis process.

## 3. Results and discussion

### 3.1. XRD analysis

**Fig. 1:**
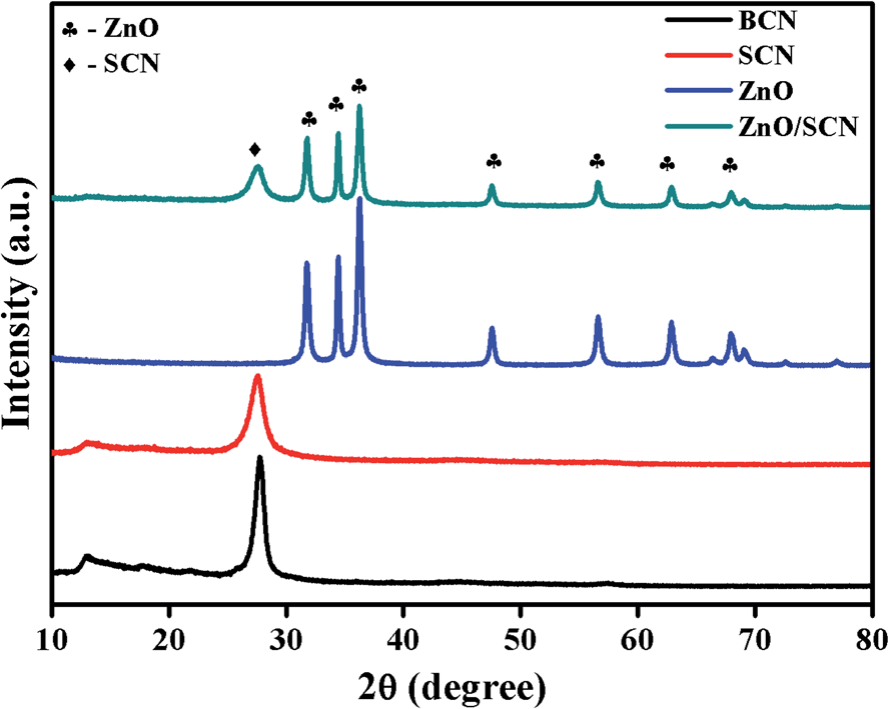
XRD patterns of pure and composite photocatalysts.

The structural and crystalline nature of the pure and composite photocatalysts was investigated through powder X-ray diffraction, and the obtained diffraction patterns are shown in Fig. 1. From the XRD pattern, it was clearly observed that the BCN and SCN have similar diffraction patterns with two distinct planes (100) and (002). The plane (100) is attributed to the in-plane repeated tri-*s*-triazine motif in the carbon nitride matrix. The diffraction plane (002), describes the interlayer distance between the conjugated aromatic rings.^13–15^ When compared to BCN, the diffraction peak intensity of the plane (002) has reduced in SCN, which demonstrated the formation of melamine during the heating of thiourea. Moreover, the peak position of the (002) in SCN shifted to a lower angle of 27.58° compared to the BCN peak position of 27.75°. This result reveals the enlargement of the stacking distance between the conjugated aromatic systems through doping with sulfur into the g-C_3_N_4_ network. Besides, the peak at 12.95° for SCN becomes weaker in comparison with bare carbon nitride, which shows the sulfur atom placed in the carbon nitride crystal lattice.^20,22,23^ Furthermore, the XRD pattern of ZnO shows the hexagonal wurtzite crystal structure which is well-matched with previous reports and JCPDS (36-1451).^6^ The diffraction peaks at 2*θ* values of 31.75°, 34.41°, 36.25°, 47.62°, 56.60°, 62.88°, 67.86°, and 69.07° were indexed as (100), (002), (101), (102), (110), (103), (112) and (201), respectively. It is observed that the diffraction patterns of SCN and ZnO were presented in the composite heterojunction photocatalyst with lower diffraction intensity. In addition to this, peak broadening also appeared in the composite photocatalysts compared to pure ZnO. The peak broadening and peak intensity reduction demonstrated the lower crystalline size of ZnO during the heterojunction formation. No other peak related to the ZnS was observed in the composite photocatalyst revealing the phase purity of the sample. There are no peak shifts observed in ZnO diffraction positions in the composite, revealing the anchoring of ZnO crystals in the SCN nanosheet.

### 3.2. UV-DRS analysis

**Fig. 2:**
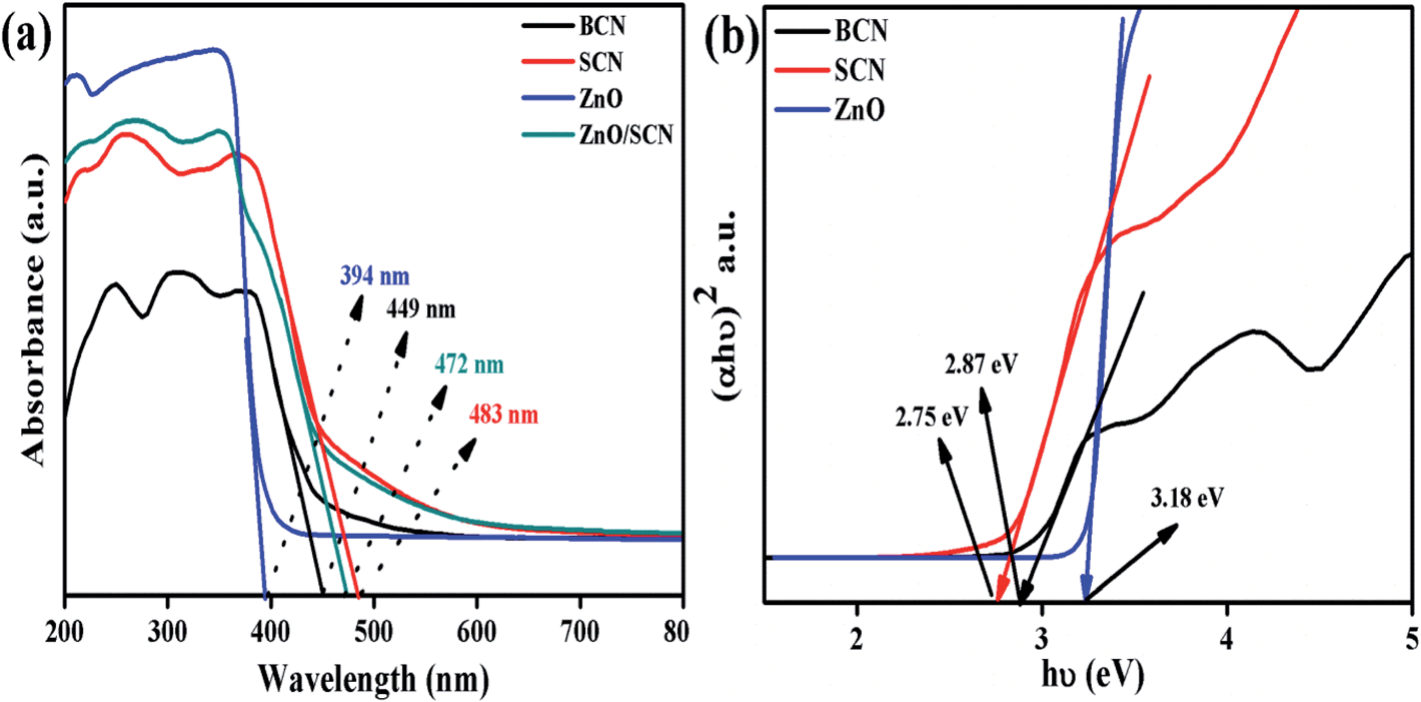
UV-vis-DRS analysis of the photocatalysts: (a) UV-vis absorption spectrum, and (b) Tauc plot.

xThe optical absorption properties of the nanoparticles were investigated using UV-vis-diffuse reflectance spectroscopy, and the absorption spectra are depicted in Fig. 2(a). It is noted that the bare carbon nitride exhibits visible light absorption at 449 nm. The typical visible light absorption of the carbon nitride revealed the charge transfer from N 2p orbitals to O 2p orbitals.^20^ It is clearly observed that the addition of sulfur into the g-C_3_N_4_ matrix improves the light absorption wavelength and increases the light absorption intensity in the UV region. The redshift in the SCN clearly described the presence of S in the carbon nitride crystal system.^22^ The measured UV-vis absorption wavelength of the SCN was 483 nm. The sulfur doping helps to harvest more solar light to produce a large number of charge carriers for an effective photocatalysis process. The pure ZnO nanoparticle shows an optical absorption wavelength at 394 nm. Furthermore, the ZnO nanoparticles show the highly dense absorption in the UV region and show transparency in the visible light region due to the electronic transition from O 2p orbitals to Zn 3d orbitals. The bandgap of the bare g-C_3_N_4_, pure ZnO, and SCN were determined by the Tauc plot as shown in Fig. 2(b). The measured bandgap values of BCN, SCN, and ZnO were 2.87 eV, 2.75 eV, and 3.18 eV, respectively. From the UV absorption spectrum of the ZnO/SCN nanocomposite, it is noted that the heterojunction exhibits a slight blue shift (478 nm) in comparison with pure SCN due to the quantum confinement effect. This is well-matched with the crystallite size reduction of ZnO nanoparticles in the composite photocatalyst system.

The direct bandgap of the material was related to the crystallite size of the nanoparticles. With lower crystallite size, the electronic structures are completely discrete which does not allow the electronic transition towards the red wavelength side. Besides they produce an electronic population in a discrete energy state which leads to an enlarged bandgap with a strong blue shift. These results conclude that the blue shift in the composite photocatalysts in comparison with SCN, is due to the quantum confinement effect.^6^ However, the composite ZnO/SCN photocatalyst shows high absorption wavelength compared to pure ZnO. The photoinduced charge carriers inside the composite photocatalyst readily transferred to the surface of the photocatalyst across the heterojunction in the interlayer direction. Hence, the optical absorption study reveals that a large amount of electron–hole pairs are generated in the composite photocatalyst system which leads to higher photocatalytic activity relative to the pure ZnO and bare SCN.

### 3.3. FTIR analysis

**Fig. 3:**
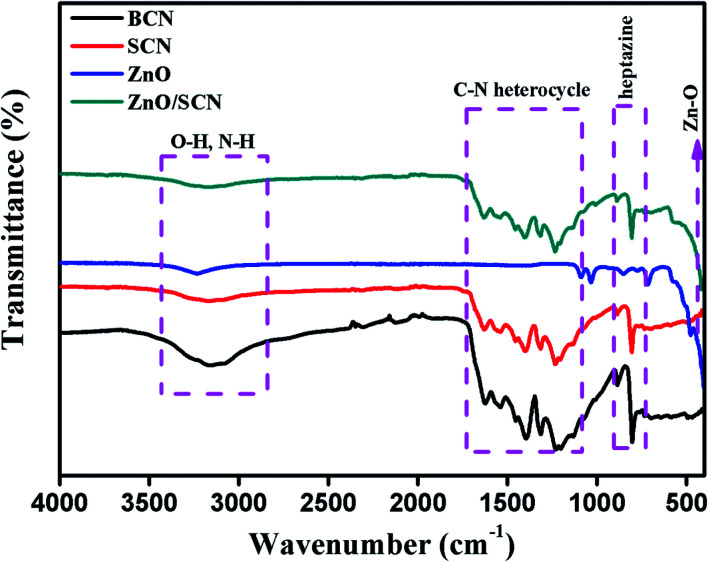
FTIR spectra of the pure and composite photocatalysts.

The functional group presented in the photocatalysts and their vibrational properties was analyzed by FTIR spectroscopy and the recorded spectra are shown in Fig. 3. The FTIR transmittance spectrum of pure BCN and SCN show similar vibrational properties. The FTIR spectrum presented in the region between 1200–1600 cm^−1^ is attributed to the C–N/C=N heterocycle in the triazine unit of the carbon nitride crystal matrix. The heptazine unit presented in the g-C_3_N_4_ was observed at a wavenumber of 811 cm^−1^ in the IR spectrum. The surface adsorbed hydroxyl group and unreacted amine group are presented in the region between wavenumbers 2900–3300 cm^−1^. No characteristic spectrum related to sulfur elements was observed so there might be a lower amounts of sulfur in the carbon nitride matrix.^14,22^ The pure ZnO exhibits an FTIR spectrum at 445 cm^−1^ assigned to Zn–O bonding.^6^ The composite ZnO/SCN photocatalysts exhibit the characteristic peaks of both SCN and ZnO nanoparticles.

### 3.4. Morphological analysis

**Fig. 4:**
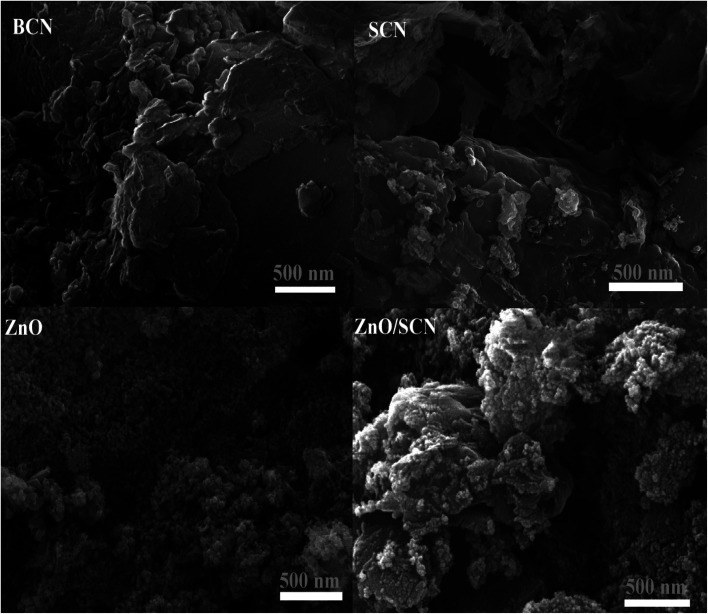
FESEM images of pure and composite photocatalysts.

**Fig. 5:**
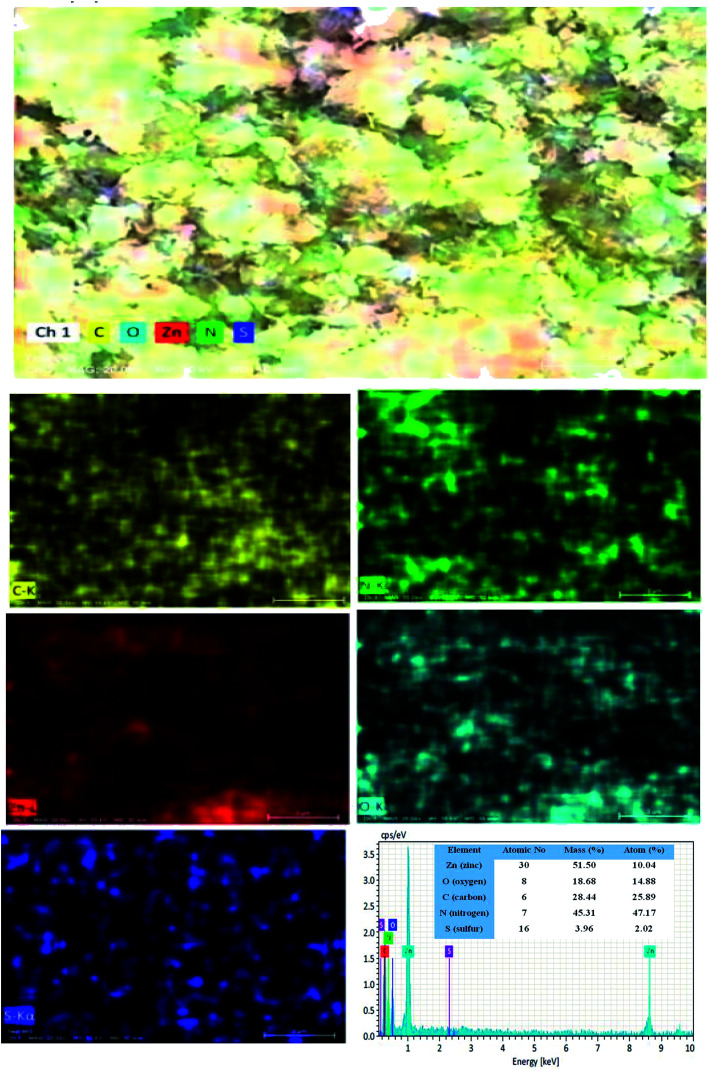
Elemental distribution and EDS analysis of ZnO/SCN nanocomposite.

The surface morphological nature of the prepared photocatalysts was analyzed by FESEM micrographs and the recorded images are shown in Fig. 4. From the FESEM micrograph, it can be seen that the prepared bare carbon nitride shows flake-like morphology. The S-doped g-C_3_N_4_ exhibits a wrinkled sheet-like morphology well-matched with a previous literature report.^24^ It is seen from the FESEM micrograph that pure ZnO has a highly agglomerated spherical-like morphology. From the FESEM image, it was noted that the aggregated ZnO nanoparticles are deposited on the surface of the SCN nanosheet in a binary ZnO/SCN composite heterojunction. The deposited ZnO nanoparticles on the SCN nanosheet produce the heterojunction, which helps to improve the photocatalytic activity. The elemental distribution and the surface element composition were further analyzed by EDS spectroscopy and the obtained EDS spectrum and elemental mapping is illustrated in Fig. 5. The elemental distribution and EDS images revealed the presence of Zn, O, C, N, and S elements in the composite photocatalyst. Moreover, the concentration of the elements presented in the ZnO/SCN composite photocatalysts is shown in Fig. 5. This result is evidence for the presence of ZnO and SCN nanoparticles in the composite photocatalyst system.

**Fig. 6:**
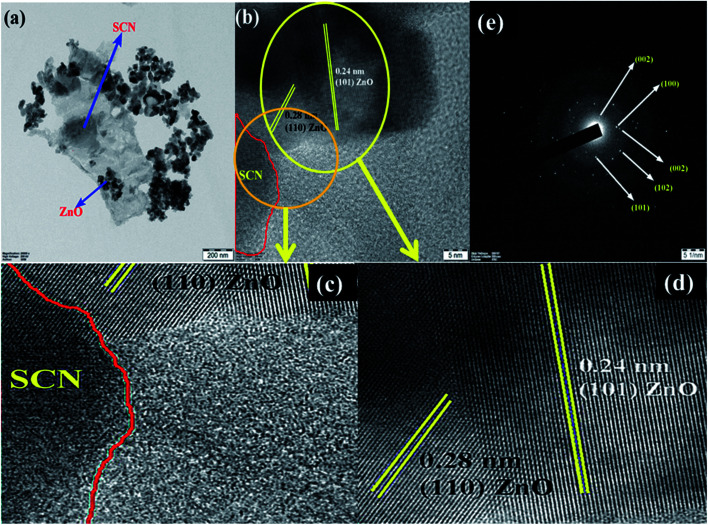
TEM analysis of ZnO/SCN: (a) TEM, (b) HRTEM, (c and d) enlarged HRTEM and (d) SAED pattern.

Furthermore, to confirm the interaction between the nanoparticles in the ZnO/SCN composite photocatalyst, TEM analysis was carried out and the results are shown in Fig. 6(a). It is obvious, that the ZnO nanoparticles were deposited on the SCN nanosheet. Moreover, the existence of the heterojunction between the nanoparticles was analyzed by HRTEM and the micrograph is shown in Fig. 6(b). From the HRTEM image, the inter-planar distance of 0.28 nm and 0.24 nm correspond to the planes (110) and (101) of ZnO nanoparticles. The smooth contact between the ZnO and SCN was detected from the HRTEM micrograph. The lattice fringes were not detected in the SCN due to the low crystallinity/thin-layered sheet-like morphology.^15^ The SAED pattern of the ZnO/SCN reflects the polycrystalline nature of the prepared composite photocatalyst. The TEM analysis proposed that the string heterojunction between the nanoparticles facilitates the improved photocatalytic behavior and helps to reduce the recombination rate of photo-induced charge carriers. Moreover, the sheet-like nature of the SCN is beneficial for electron transport behavior across the interlayer heterojunction between the nanoparticles, and is helpful for increasing the lifetime of the photo-excited charge carriers for effective photocatalytic activity.

### 3.5. Surface chemical analysis

**Fig. 7:**
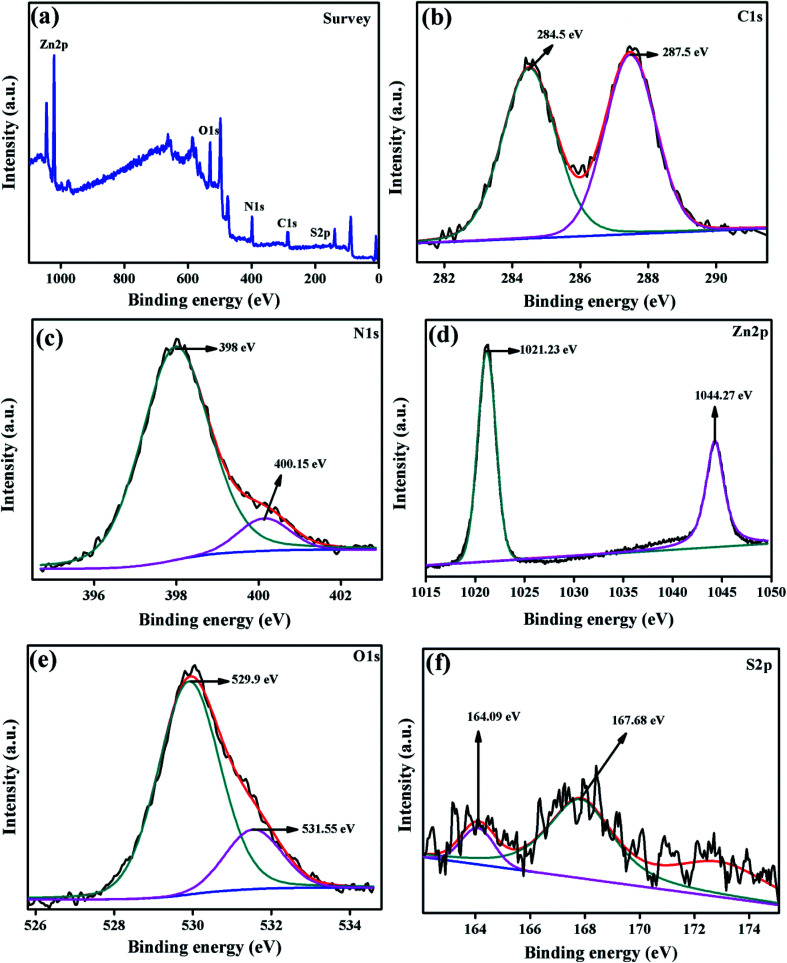
XPS spectrum of the ZnO/SCN composite photocatalyst.

The chemical composition and elements presented in the surface of the composite photocatalyst were examined by XPS analysis and the spectrum is displayed in Fig. 7. The survey spectrum of ZnO/SCN elucidated the presence of carbon, nitrogen, oxygen, zinc, and sulfur elements in the photocatalyst. The high-resolution peak with the deconvolution of all the elements is displayed in Fig. 7(b–f). The C 1s high-resolution spectrum deconvoluted into two distinct peaks positioned at 284.5 eV and 287.5 eV attributed to the graphitic C–C and C–N bonding in g-C_3_N_4_. The nitrogen deconvoluted high-resolution spectrum is shown in Fig. 7(c). The deconvoluted peaks positioned at the binding energy of 398 eV and 400.15 eV reveal the C=N–C bonding and ternary nitrogen bonded carbon in the triazine unit (N–C_3_). The deconvoluted Zn 2p spectrum shows the presence of Zn 2p_3/2_ and Zn 2p_1/2_ peaks with binding energy of 1021.23 eV and 1044.27 eV, respectively. The presence of zinc ions with the oxidation value of +2 was confirmed by the Zn 2p spectrum. The O 1s deconvoluted peaks at a binding energy of 529.9 eV and 531.55 eV are attributed to the metal–oxygen bonding in the ZnO crystal system with an oxidation state of O^2−^, and surface adsorbed OH molecules, respectively.^13,15^ The S 2p high-resolution deconvoluted spectrum is shown in Fig. 7(f). The peak at the binding energy of 164.09 eV described the S–C bonding in the SCN matrix, and the peak at 167.68 eV described the sulfate with an oxidation state of S^2−^.^22^ Hence, the XPS spectrum of the ZnO/SCN nanocomposite disclosed a strong chemical interaction between the nanoparticles, which leads to the heterojunction formation.

### 3.6. Photoluminescence analysis

**Fig. 8:**
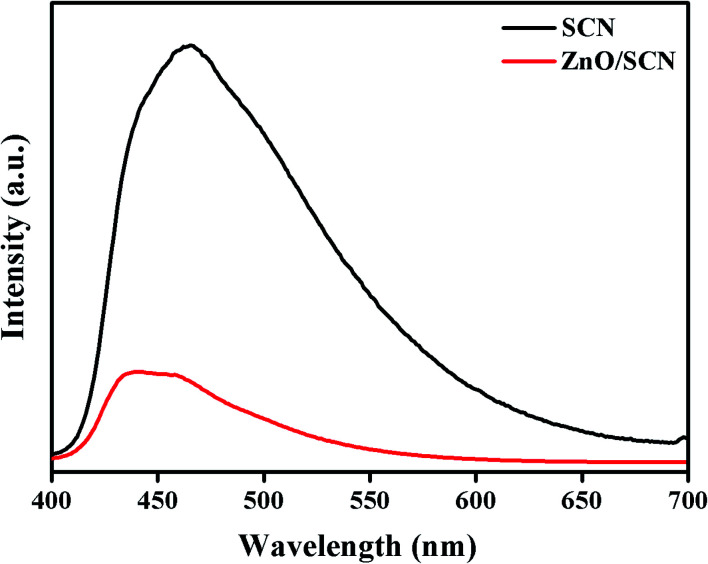
Photoluminescence spectrum of SCN and ZnO/SCN.

To confirm the electron–hole recombination rate, electron trapping, and photo-induced charge carrier separation in the prepared photocatalysts, the PL analysis was carried out and the emission spectrum is displayed in Fig. 8. The PL emission spectrum for photocatalysts was recorded at room temperature with excitation of 390 nm. The high intensity peak reveals a large amount of electron–hole recombination. As observed from the PL emission spectrum, the peak intensity of the SCN is much higher than the ZnO/SCN nanocomposite. The PL spectrum of pure SCN exhibits the emission peak positioned at a wavelength of 470 nm, which is well-matched with the UV-vis-DRS result. It is noted that the peak intensity decreased in ZnO/SCN which clearly described the low recombination rate of photo-induced charge carriers. Besides, the emission peak of the ZnO/SCN composite slightly shifted to the blue wavelength side which is attributed to the defect trap state due to the quantum confinement effect, and it is well-matched with the UV-vis-DRS absorption study.^13,20^ From the PL analysis, one can conclude that the heterojunction between the SCN and ZnO nanoparticles encourages the interfacial charge transport and effective charge separation. Hence, the composite photocatalyst is an efficient candidate for photocatalytic organic pollutant removal under LED light irradiation.

### 3.7. Photocatalytic activity

**Fig. 9:**
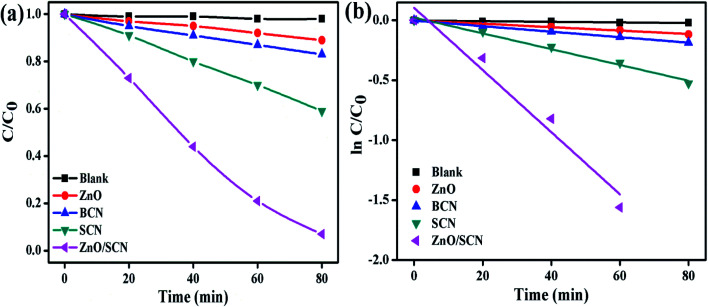
(a) MB degradation, and (b) 1^st^ order kinetic plots of the pure and composite photocatalysts.

The MB and RhB were used as model pollutants to investigate the photocatalytic degradation efficiency of the prepared nanomaterials. The commercial indoor LED lamp was employed as a visible light source for the photocatalytic degradation process. The dye concentration/degradation efficiency *vs.* irradiation time and first-order kinetic plot for MB dye solution, are displayed in Fig. 9. From the degradation plot, it was observed that the MB dye solution was stable and shows negligible degradation in the photolysis process. This result reveals the stability of the dye molecules against the degradation of environmental conditions. From the MB dye degradation plot, it can be concluded that the composite photocatalyst shows the best degradation efficiency when compared to pure nanomaterials. The dye degradation efficiency of 93% was attained after 80 min of LED light illumination using the ZnO/SCN composite photocatalyst. The recorded degradation efficiency of pure ZnO, BCN, and SCN under visible light irradiation was 11%, 17%, and 41%, respectively. The pure ZnO exhibits very low degradation efficiency compared to other nanoparticles. This degradation result reveals that the LED light emits a very low portion of the UV-light in addition to the visible light. Hence, the pure ZnO generates electron–hole pairs under UV radiation and contributes to the photocatalytic degradation process. The reason for the very low photocatalytic degradation efficiency of the ZnO is as follows: (i) the production of a very low amount of charge carriers due to the lesser portion of UV light in the LED light spectrum, and (ii) fast photo-induced electron–hole recombination rate.^31^ The enhanced dye degradation efficiency of the composite photocatalysts is mainly attributed to the superior visible light absorption ability and effective separation of photon generated electron–hole pairs. The 1^st^ order reaction rate constant for dye degradation under LED light irradiation was calculated using the relation^13^ln(*C*/*C*_0_) = −*kt*

The rate constant value of the ZnO/SCN nanocomposite photocatalyst was 0.0259 min^−1^, which is 20, 12, and 5 times higher than the pure ZnO (0.0014 min^−1^), BCN (0.0023 min^−1^) and SCN (0.0066 min^−1^) photocatalysts, respectively.

**Fig. 10:**
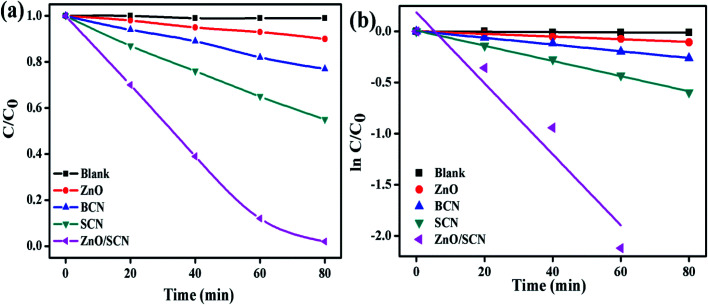
(a) RhB degradation, and (b) 1^st^ order kinetic plots of the pure and composite photocatalysts.

Furthermore, the degradation ability of the prepared nanoparticles was analyzed by the degradation of RhB dye solution under ambient environmental conditions like MB dye degradation. The RhB dye concentration with respect to the LED light irradiation plot is depicted in Fig. 10(a). The photolysis process clearly described the stability of RhB under light illumination and it shows the negligible amount of degradation. The maximum dye degradation efficiency of 98% was observed for the ZnO/SCN composite photocatalyst under visible light irradiation. The calculated degradation efficiency of the SCN nanoparticle was 45%, which is 4.5 and 2 folds higher than the pure ZnO and bare BCN, respectively. The kinetic rate plot and *k* value of pure and composite photocatalysts are shown in Fig. 10. The calculated rate constant value of RhB dye degradation for the ZnO/SCN nanocomposite was 0.0347 min^−1^ after 80 min of LED light illumination. The calculated rate constant values of 0.0013 min^−1^, 0.0033 min^−1^, and 0.0074 min^−1^ are attributed to pure ZnO, BCN, and SCN photocatalysts, respectively. From the photocatalytic report, we can conclude that the composite heterojunction shows the maximum degradation efficiency due to the higher visible light absorption and lower photon generated electron–hole recombination.

**Fig. 11:**
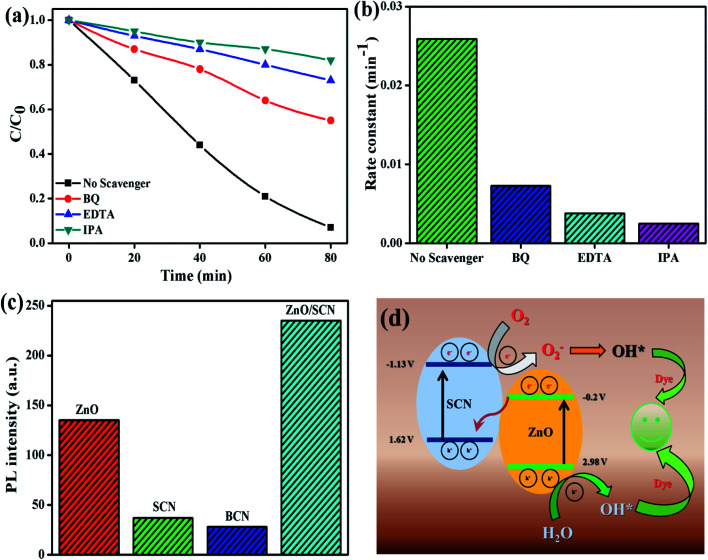
(a and b) Elemental trapping experiment, (c) TA test, and (d) reaction mechanism.

To identify the reactive species for the photocatalytic process, the scavengers test has been conducted for MB dye degradation and the results are shown in Fig. 11(a and b). From the trapping experiments, it has been clearly observed that hydroxyl radicals are the main reactive species for the degradation process. Moreover, EDTA and BQ also play a minor role in the degradation reaction. The calculated MB dye degradation efficiency of 18%, 27%, and 45% correspond to the scavengers IPA, EDTA, and BQ, respectively. The kinetic rate constant value for EDTA, BQ, and IPA was 0.0038 min^−1^, 0.0073 min^−1^, and 0.0025 min^−1^, respectively. The elemental trapping experiment demonstrated that the ˙OH radicals are the principal radicals for the dye degradation process under LED light irradiation. Furthermore, the production of ˙OH radicals was determined by the TA test. The fluorescent HTA (hydroxyl terephthalic acid) was produced when TA reacted with ˙OH radicals. This HTA production was monitored by PL emission at 425 nm with an excitation wavelength of 315 nm. The PL intensity increases with increasing production rate of ˙OH radicals during the LED illumination. From Fig. 11(c), we can conclude that the composite ZnO/SCN photocatalyst produces the maximum ˙OH radicals in comparison with pure photocatalysts. Hence, the TA test also confirms the production of hydroxyl radicals mainly due to the low charge carrier recombination rate and high visible light absorption ability of the composite photocatalyst.^28^

Based on the elemental trapping experiment and TA test the possible reaction mechanism was proposed and is illustrated in Fig. 11(d). It is noteworthy to determine the band potential of the photocatalysts to propose the reaction mechanism. The conduction and valance band potential was calculated using the relation^15^*E*_CB_ = *χ* − *E*_e_ − 0.5*E*_g_*E*_VB_ = *E*_CB_ + *E*_g_where, *χ*, *E*_e_ and *E*_g_ denote the electronegativity, electron energy in hydrogen scale (4.5 eV), and bandgap, of the photocatalysts. The calculated CB values are −1.13 V (in V *vs.* NHE) and −0.2 V for SCN and ZnO nanoparticles, respectively. According to the valance band relation, the calculated value of 1.62 V and 2.98 V are assigned to the SCN and ZnO photocatalysts, respectively.

The predicted possible photocatalytic reaction mechanism is as follows; when the LED light is irradiated on the surface of the photocatalysts the electrons are excited and transferred to the CB of both catalysts. Then the CB electrons at SCN are transferred to the CB of ZnO. Meanwhile, the holes are transferred to the valence band (VB) of SCN from the VB of ZnO in a type-II heterojunction. But the VB potential of SCN and CB potential of ZnO is not sufficient enough to initiate the photocatalytic redox process due to the low potential compared to standard oxidation (1.99 V *vs.* NHE) and reduction (−0.33 V *vs.* NHE) potential. However, the elemental trapping experiment shows that the superoxide and holes contributed to the photocatalysis process. Moreover, the hydroxyl radicals are the key factor for the degradation reaction. Based on these results we have proposed the Z-scheme photocatalysis mechanism for the degradation process. In this Z-scheme process, the electron at ZnO CB was migrated to the VB of SCN through interfacial contact in the heterojunction, and was excited to the CB of SCN. On the other hand, the holes accumulated at the VB of the ZnO nanoparticles. In this way, the electrons and holes are separated in opposite directions in the photocatalyst heterojunction. This interfacial charge transformation/recombination leads to the electron concentration at SCN and holes concentration at ZnO with a considerably high reduction potential (−1.13 V *vs.* NHE) and high oxidation potential (2.98 V *vs.* NHE), respectively. These localized charge carriers can drive the reduction and oxidation reaction, which can be experimentally examined by elemental trapping experiments and the terephthalic acid test.^29^ The accumulated electrons at the CB of SCN reduce the oxygen to superoxide radicals, while VB holes at ZnO oxidize the water into ˙OH radicals. The produced O_2_^−^ radicals further generate the ˙OH radicals by reacting with H_2_O. In this way, the ˙OH radicals production rate is enhanced by the heterojunction photocatalyst during the photocatalysis process. The highly generated ˙OH radicals degrade the dye molecule with the liberation of water and carbon dioxide. In this way, the charge carrier transportation across the interfacial contact between the heterojunction in a Z-scheme photocatalyst system is physically more possible than the conventional type-II heterojunction, since the migration of the photo-induced electrons from the CB of the ZnO to the photon-generated hole rich VB of the SCN is favorable due to the electrostatic attraction between the electron and hole. Whereas, in type-II heterojunction photocatalysts the electron and hole migration from the CB of SCN to the CB of ZnO and hole migration from the VB of ZnO to the VB of SCN was restricted due to the electrostatic repulsion between electron–electron or hole–hole. On the other hand, the light-shielding effect caused by the electron (donor/acceptor) mediator can also be reduced by this type of direct Z-scheme photocatalyst system.^30^ The possible photocatalytic reaction mechanism for dye degradation is as follows^8,15^ZnO/SCN + *hν* → ZnO (e^−^/h^+^) + SCN (e^−^/h^+^)ZnO (e^−^) → SCN (VB)ZnO (h^+^) + H_2_O → ˙OH + H^+^SCN (e^−^) + O_2_ → O_2_^−^O_2_^−^ + H^+^ + e^−^ → OH^−^ + ˙OH˙OH + dye → intermediate products

### 3.8. Recycle test

**Fig. 12:**
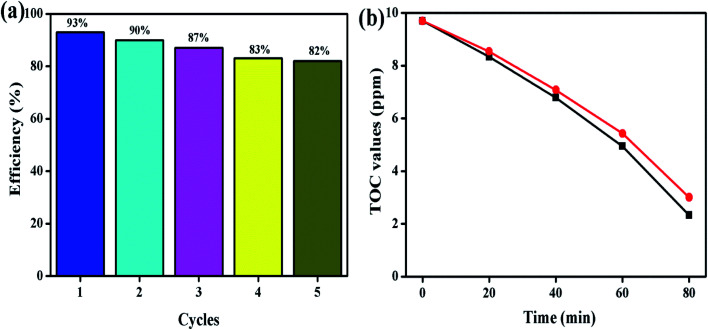
(a) Recycle test, and (b) TOC removal analysis.

To identify the stability of the prepared photocatalyst, the recycle test was conducted and the obtained result is shown in Fig. 12. The recycle test was conducted on the MB dye solution under LED light illumination at an ambient atmosphere. Five successive recycle tests were conducted for MB dye degradation using the ZnO/SCN composite photocatalyst. The composite photocatalysts exhibit 80% degradation efficiency even at the 5^th^ cycle, which reflects the stability of the prepared nanoparticle. Moreover, the total organic carbon analysis was performed to determine the removal percentage of carbon content from the MB dye solution during the photocatalysis process. The TOC removal percentage for MB dye solution degradation using ZnO/SCN nanoparticles was 76% and 69%, as observed at the initial and final cycle, respectively in Fig. 12(b). Hence, we can conclude that the prepared composite ZnO/SCN photocatalyst is a stable material for practical use in photocatalytic degradation processes.

## 4. Conclusion

In conclusion, the simple sol–gel process was used for synthesizing effective composite photocatalysts. The prepared ZnO embedded SCN heterojunction composite shows enhanced optical properties and charge separation behavior, which was thoroughly analyzed by UV-vis-DRS and PL spectroscopy. Under LED light irradiation the composite photocatalyst shows maximum dye degradation efficiency compared to pure ZnO and pristine SCN nanoparticles, due to the synergistic effect between the nanoparticles. The improved photocatalytic behavior of the nanocomposite reveals the high rate of ˙OH radicals production through oppositely separated charge carriers, which was confirmed by the TA test and trapping experiment. Moreover, the recycle test confirms the stability of the photocatalysts. Hence, the prepared nanocomposite ZnO/SCN is a promising catalyst for environmental remediation applications.

## Conflicts of interest

The authors declare that they have no conflicts of interest.

## Supplementary Material
